# Repairable, Degradable and Recyclable Carbon Fiber-Reinforced Bio-Based Epoxy Vitrimer Composites Enabled by Facile Transesterification

**DOI:** 10.3390/polym17172387

**Published:** 2025-08-31

**Authors:** Haidan Lin, Kai Dong, Jingyao Luan, Chenggang Li, Di Zhao, Chengji Zhao, Xuefeng Li

**Affiliations:** 1 Key Laboratory of High Performance Plastics, Ministry of Education, College of Chemistry, Jilin University, Changchun 130012, China; dilys0121@163.com (H.L.); dongkai24@mails.jlu.edu.cn (K.D.); dzhao1123@163.com (D.Z.); zhaochengji@jlu.edu.cn (C.Z.); 2 Electric Power Research Institute, State Grid Jilin Electric Power Co., Ltd., Changchun 130012, China; yijian1105@163.com (J.L.); lcgdky@126.com (C.L.)

**Keywords:** bio-based epoxy vitrimer, carbon fiber-reinforced composites, chemical degradation, recovery, sustainability

## Abstract

Developing high-performance bio-based epoxy resins as sustainable alternatives to petroleum-derived bisphenol A (BPA) epoxies for recyclable carbon fiber-reinforced polymers (CFRPs) is pivotal in materials research. Herein, the bio-based bisphenol monomer BDEF was synthesized from the lignin derivative 4-propylguaiacol. The derived epoxy monomer BDEF-EP was cured with adipic acid to form a bio-based vitrimer. Stress relaxation synergistically accelerates through intrinsic dynamic carboxylic acid ester exchange and enhanced chain mobility from the flexible propyl structure. At 220 °C, this vitrimer shows rapid stress relaxation (τ* < 30 s) and repairs ~90% of surface scratches in 30 min. It exhibits tensile and flexural strengths of 69 MPa and 105 MPa, respectively. BDEF-EP’s low viscosity reduces diluent needs in composite fabrication, lowering costs and improving efficiency. The resulting bio-based CFRP achieves tensile and flexural strengths of 543 MPa and 414 MPa, respectively, which are comparable to commercially available petroleum-derived CFRP. In addition, CFRP containing dynamic crosslinked networks demonstrates degradable recyclability in ethylene glycol solvent, preserving the surface morphology and chemical structure of recovered carbon fibers. The results demonstrate that this bio-based epoxy vitrimer has promising potential for developing sustainable, degradable, and recyclable CFRP composites.

## 1. Introduction

Epoxy resins are the most widely used thermosetting polymers in modern industry, owing to their exceptional mechanical strength, chemical resistance, and interfacial adhesion. These thermosetting resins are extensively applied in aerospace composites, electronic encapsulation, anticorrosion coatings, and structural adhesives [1,2,3]. Specifically, their carbon fiber-reinforced polymer (CFRP) composites featuring an exceptional strength-to-weight ratio and corrosion resistance have been widely employed in high-performance structural components across multiple industries, including aerospace, automotive, renewable energy, and sports equipment [4,5]. However, conventional petroleum-based epoxy resins face significant challenges in chemical degradation and recycling due to their permanent irreversible thermosetting crosslinked networks. Petroleum-based epoxy resins, a significant category of traditional polymer materials, face growing resource sustainability challenges. The global annual demand for epoxy resins exceeds 3 million tons. Over 90% rely on petroleum-based feedstocks, with BPA constituting up to 75% of primary raw materials. This heavy reliance creates significant vulnerability to petroleum supply fluctuations. Moreover, petroleum’s non-renewable nature and price volatility critically constrain industry development. Resource bottlenecks for petroleum-based epoxy resins manifest in three aspects: (1) supply chain vulnerabilities magnify raw material instability; (2) structural oversupply risks in synthetic resins lead to inefficient resource utilization; (3) BPA’s reproductive toxicity and environmental persistence prompt stricter global regulations. Upon end-of-life structural degradation or irreversible damage, they are unsuitable for reuse, requiring disposal through environmentally detrimental methods, such as landfilling and incineration, which usually result in greenhouse gas emissions, toxic byproducts, and persistent microplastic pollution [6].

Furthermore, conventional petroleum-based epoxy resins are primarily synthesized from bisphenol A (BPA), a petroleum-derived precursor classified as an endocrine-disrupting chemical [7]. Safety concerns regarding BPA-derived epoxy resins have emerged, as studies indicate that BPA leaching can induce estrogenic activity, which has been associated with reproductive disorders and metabolic syndrome [8,9]. These concerns have prompted regulatory actions, including restrictions and bans by the European Union and the U.S. Food and Drug Administration. In addition to health risks, the production and application of petroleum-derived BPA pose significant environmental and resource-related challenges. Naturally occurring phenolic compounds derived from biomass offer promising alternatives for addressing sustainability challenges and developing novel low-toxicity monomers [10,11,12]. The inherent aromatic ring structures and abundant availability of plant polyphenols provide robust material platforms for synthesizing bio-based compounds containing heterocyclic, aliphatic, and aromatic systems, serving as sustainable alternatives to petroleum-derived BPA in epoxy resin production. Representative aromatic renewable resources, such as eugenol, guaiacol, ferulic acid, and vanillin, demonstrate significant potential for application in the development of novel chemicals and polymers as sustainable feedstocks [13].

Recent advances in bio-based epoxy resins have enabled the development of epoxy vitrimers that incorporate adaptive covalent networks for CFRP composites [14,15]. These materials retain thermoset-like dimensional stability under service conditions, while demonstrating thermoplastic-like reprocessability through thermally triggered bond exchange mechanisms. At temperatures exceeding the topology freezing transition temperature (*T*_v_), rapid dynamic covalent exchange reactions facilitate stress relaxation via dynamic network reconfiguration, effectively transforming the crosslinked matrix into a viscoelastic fluid. Therefore, the resulting epoxy vitrimers can be reprocessed, degraded, and recycled through dynamic bond exchange above *T*_v_, while preserving mechanical integrity under *T*_v_ [16]. Leibler et al. first reported the preparation of epoxy vitrimers via an epoxy-anhydride reaction system [15]. At elevated temperatures, ester exchange reactions induce the topological rearrangement of dynamic crosslinking networks, imparting malleability and recyclability to epoxy vitrimers [17,18,19]. Recent research increasingly focuses on incorporating multiple dynamic bonds into polymer networks, leveraging their unique advantages to develop multi-responsive vitrimers. However, reversible dynamic covalent bonds inherently trade network stability for topological adaptability, often resulting in reduced mechanical strength. For example, Zhang et al. synthesized epoxy vitrimers through simultaneous disulfide metathesis and ester exchange. Although this approach enabled efficient reprocessing at lower temperatures, the low bond energy of disulfide bonds led to diminished mechanical strength after multiple cycles [18]. Liu et al. also incorporated disulfide bonds and ester exchange catalysts into epoxy vitrimers. The results showed that increasing the disulfide bond content enhanced the dynamic properties but concurrently reduced their mechanical strength [20]. Therefore, enabling the efficient topological rearrangement of dynamic crosslinking networks without significantly compromising mechanical stability remains challenging in the molecular design of epoxy vitrimers.

The development of novel bio-based epoxy resins to replace traditional BPA-based types has become a research hotspot in materials science. This interest stems from concerns regarding BPA, an endocrine disruptor with associated health and environmental risks, and the reliance of conventional epoxy resins on non-renewable petroleum resources. In response to these challenges, research efforts have focused on dynamic epoxy resins that incorporate dynamic carboxylate bonds to form a crosslinked network. This design aims to retain the inherent strength of the material while introducing excellent dynamic properties. The performance of these novel resins will be comprehensively evaluated through comparison with commercial BPA-based epoxy resins. In this work, we reported a bio-based carboxylic acid-cured epoxy vitrimer derived from the lignin derivative 4-propylguaiacol and cured with commercial adipic acid (AA). The incorporation of a flexible propyl side chain can significantly reduce the viscosity of the epoxy monomer, facilitating vitrimer processing and enhancing composite molding efficiency. Furthermore, the flexible propyl side chains, in synergy with dynamic ester bonds, facilitated chain migration and enhanced the dynamic properties of the bio-based vitrimers without incorporating multiple dynamic bonds, thereby preserving the intrinsic properties of epoxy resins. Specifically, compared to the control BPA-based epoxy vitrimer (E51-AA), the flexible propyl group significantly enhanced the dynamic properties of the dynamic crosslinked network. Moreover, its CFRP composites fabricated via vacuum resin infusion exhibited excellent mechanical properties. Specifically, the vitrimer matrix can be effectively degraded in ethylene glycol, enabling the gentle recycling of high-quality carbon fibers from its CFRP composites. The results indicate the bio-based epoxy vitrimer has promising potential for developing sustainable, degradable, and recyclable CFRP composites.

## 2. Materials and Methods

### 2.1. Materials and Reagents

4-Propyl-2-methoxyphenol, tetra-n-butyl-ammonium bromide (TBAB), adipic acid (AA), and zinc acetylacetonate hydrate (Zn(acac)_2_) were purchased from Shanghai Aladdin Biochemical Technology Co., Ltd., Shanghai, China. Methyl aldehyde, epichlorohydrin (ECH), vanillin, and phosphoric acid were supplied from Energy Chemical Co., Ltd., Shanghai, China. Acetone, toluene, tetrahydrofuran (THF), ethylene glycol (EG), petroleum ether, and N, N-dimethylformamide (DMF) were received from Sinopharm Chemical Reagent Co., Ltd., Shanghai, China. All materials were used as received without further purification. A plain weave T300-1K carbon fiber cloth was obtained from Toray, Japan. Bisphenol A diglycidyl ether (tradename: E-51) with an epoxy equivalent of 196 g eq^−1^ (industrial grade) was purchased from Shanghai Aotun Chemical Technology Co., Ltd., Shanghai, China.

### 2.2. Preparation of Epoxy Vitrimer and Carbon Fiber-Reinforced Composite

**Figure 1:**
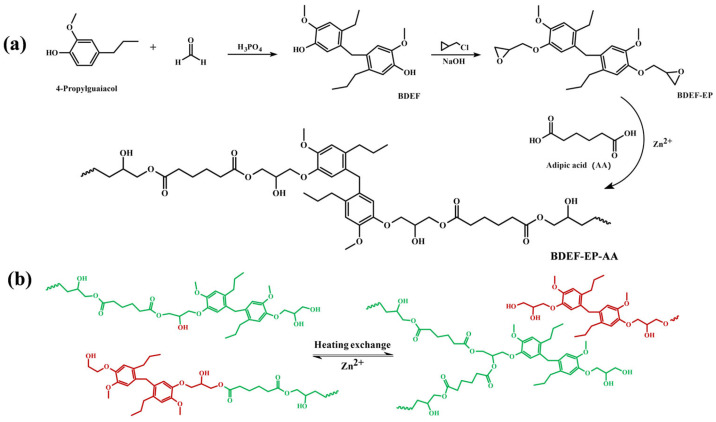
(**a**) Synthesis of the bio-based epoxy (BDEF-EP) and a schematic diagram of the BDEF–EP–AA structural unit; (**b**) curing reaction of BDEF-EP with AA and illustration of transesterification reactions in the presence of Zinc catalyst.

The synthesis of epoxy monomer (BDEF-EP) was carried out in two steps, as illustrated in Figure 1a. In the first step, 0.22 mol of 4-propyl guaiacol was mixed with 7 mL of 85% phosphoric acid. The mixture was heated to 50 °C and stirred for 0.5 h. Subsequently, 0.1 mol of formaldehyde was added, and the reaction was heated to 90 °C for 6 h. The resulting crude product was washed repeatedly with deionized water until the filtrate reached a neutral pH. It was then recrystallized from toluene. Further purification by column chromatography, followed by drying under vacuum at 50 °C for 12 h, afforded BDEF in 50% yield. In the second step, a mixture of 0.1 mol of BDEF and 0.2 g of TBAB in 160 mL of ECH was heated to 90 °C for 6 h. The unreacted ECH was removed by distillation under reduced pressure. Subsequently, 200 mL of toluene and 32 g of a 30 wt.% aqueous sodium hydroxide solution were added. The resulting mixture was heated to 90 °C for 3 h. The crude product was washed with distilled water until the aqueous layer was neutral. The organic layer was then dried, and the toluene and water were removed by distillation under reduced pressure. Finally, purification by column chromatography yielded the white solid product, BDEF-EP, at 92% yield. The detailed synthetic procedure has been reported previously [21].

The composition of epoxy vitrimer BDEF-EP-AA is provided in Table S1. BDEF-EP was melted at 120 °C, followed by the addition of stoichiometric amounts of the curing agent AA and Zn(acac)_2_ with thorough mixing to achieve homogeneity. The mixture was degassed for 10 min and transferred into preheated molds. Samples were cured sequentially at 120 °C for 4 h and 160 °C for 4 h. Parallel preparations were carried out to produce E51-AA control specimens. E51 was heated to 120 °C, followed by the addition of stoichiometric amounts of curing agent AA and Zn(acac)_2_. The mixture was then stirred thoroughly until homogeneous. The mixture was degassed for 10 min and subsequently transferred to a preheated mold. The sample was cured at 120 °C for 4 h and then at 160 °C for 4 h to obtain the final epoxy vitrimer E51-AA.

The CFRP composite laminates were fabricated via the hand lay-up method and vacuum infusion molding (VIM). For the hand lay-up method, the precursor resin and six layers of T300-1K carbon fiber woven fabric (150 × 150 mm^2^) were aligned layer-by-layer. The resulting composite exhibited a fiber volume fraction of 60%. The laminated stack was vacuum-sealed and subjected to the aforementioned curing cycle under 10 MPa. The VIM process employed a polished steel mold containing three layers of unidirectional CFs, maintaining the same fiber volume fraction. A flow distribution system comprising deflector mesh, spiral tubes, vacuum ports, and resin inlets was sequentially positioned, followed by vacuum bag sealing using specialized adhesive tapes. The preheated precursor was infused under vacuum, and followed by the curing procedure. The cured composite laminates were mechanically demolded, edge-trimmed, and measured with the uniform thickness for all specimens.

### 2.3. Characterization

#### 2.3.1. Chemical Characterization

The Nicolet iS10 (Thermo Fisher Scientific Inc., Waltham, MA, USA) was used to obtain the Fourier-transform infrared spectra (FT-IR) of the monomers and specimens using the ATR pattern. Raman spectra were recorded on a RM1000 Raman spectrograph (Renishaw, Wotton-under-Edge, UK). The laser power at the sample surface was maintained below 10.0 mW for 514 nm radiation. The scanning wavenumber range was set from 100 cm^−1^ to 3200 cm^−1^.

#### 2.3.2. Viscosity Characterization

Viscosity was measured at 30 °C on the DHR-2 rheometer (Waters Corporation, Milford, MA, USA). A 25 mm diameter parallel plate fixture with a fixed gap of 800 μm was selected, and the testing temperature was 30 ± 0.5 °C. Unidirectional steady-state shear rate scans were carried out over a range of 1000 to 0.1 s^−1^, with eight data points recorded per order of magnitude.

#### 2.3.3. Thermomechanical Characterization

The non-isothermal DSC study was performed using a DSC Q20 instrument (TA instruments, New Castle, DE, USA). Specimens (5–10 mg) were placed into an aluminum crucible and subjected to dynamic DSC analysis at a temperature range from 40 °C to 450 °C at heating rates of 5, 10, 15, 20, and 25 K min^−1^ under N_2_ atmosphere, utilizing an empty crucible as a reference. The Kissinger–Ozawa–Crane method was used to determine the reaction activation energy (*E*_a_) and nth order for the curing process.

The thermal stability was determined by thermogravimetric analyses (TGA) on a Mettler Toledo TGA instrument (Zurich, Switzerland) from 80 °C to 600 °C at a heating rate of 10 °C min^−1^ under a nitrogen flow of 20 mL min^−1^. Prior to testing, specimens were dried at 120 °C for 2 h to remove moisture and then cooled to ambient temperature.

The dynamic mechanical properties were carried out using a DMA Q850 (TA instruments, New Castle, DE, USA) in single cantilever mode with a preload force of 0.01 N and a frequency of 1 Hz. Data were recorded from 30 °C to a target temperature at a heating rate of 3 °C min^−1^. Stress relaxation curves were obtained at various temperatures using the same instrument, using 0.01 N of prestress to ensure straightness and a fixed 1% deformation. Parallel testing of five samples was performed, and data are presented as mean ± SD from two independent experiments.

#### 2.3.4. Mechanical Characterization

Dumbbell-shaped samples were subjected to tensile tests using an electronic universal testing machine (AG-I, Shimadzu, Kyoto, Japan) at a crosshead speed of 2 mm min^−1^. The bending properties were evaluated via three- or four-point bending tests on straight specimens to generate characteristic stress fields in the central region. For thin-plate tensile testing, specimens were clamped at both ends and friction-loaded to establish uniform stress fields within the gauge length. Parallel testing of five samples was performed, and data are presented as mean ± SD from two independent experiments.

#### 2.3.5. Self-Repairing Characterization

The self-repairing capacity of epoxy vitrimer was investigated by observing the evolution of surface cracks using a CNOPTEC polarized light microscope, Chongqing, China. Thin sheets of specimens were prepared, and scratches were introduced on the surface with a razor blade. The changes in scratch width were monitored at 200 °C for different heating durations. The repair efficiency was calculated using the following formula:

$$\xi =\frac{W_{1}-W_{0}}{W_{0}}\times 100\%$$

where *W*_1_ is the width of cracks after heating, and *W*_0_ is the width of the original cracks.

#### 2.3.6. Morphological Characterization

The surface morphology was observed by scanning electron microscopy (SEM, Nova nano 450, FEI Inc., Hillsboro, OR, USA) with an accelerating potential of 10 kV. The surface treatment of the material was performed by gold spraying before testing.

#### 2.3.7. Solvent Resistance

Uniform epoxy vitrimer specimens were immersed in various solvents, including methanol (MeOH), ethanol (EtOH), acetone (AC), N, N-dimethylformamide (DMF), tetrahydrofuran (THF), water (H_2_O), 1 M HCl, and 1 M NaOH, at room temperature for 7 days to preliminarily assess their solvent resistance. Subsequently, solvent resistance was evaluated by treating the specimens with ethylene glycol at 160 °C for 5 h.

#### 2.3.8. Chemical Recyclability

Building on bio-based vitrimer degradation research, CFRP specimens were immersed in ethylene glycol degradation solvent under identical conditions. After 5 h at 160 °C, complete resin dissolution enabled carbon fabric separation. Recovered fibers underwent repeated ethanol washing and drying at 50 °C until constant mass was reached.

## 3. Results and Discussion

### 3.1. Curing Kinetic Analysis

**Figure 2:**
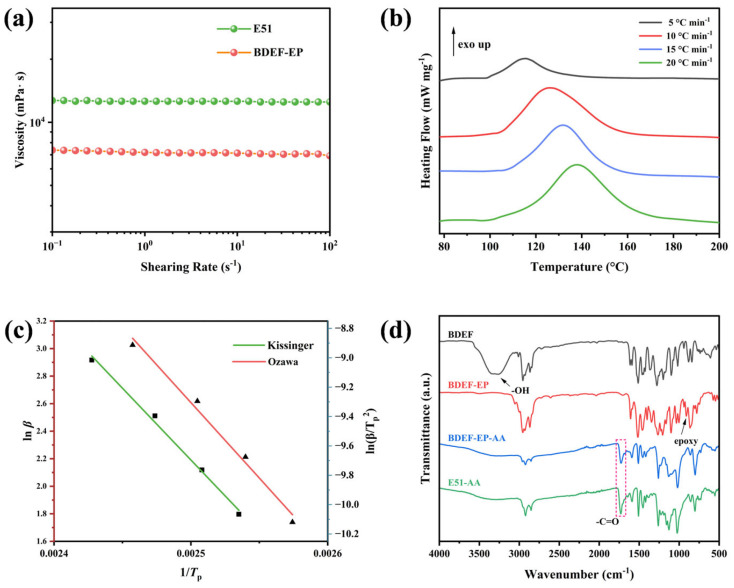
(**a**) Viscosity of E51 and bio-based epoxy monomer as a function of shear rate; (**b**) DSC curves at different heating rates; (**c**) activation energy calculated from the Kissinger and Ozawa equations; (**d**) the FT-IR spectra of BDEF, BDEF-EP, BDEF-EP-AA, and E51-AA in the range of 4000–500 cm^−1^.

The flexible propyl side chain in 4-propylguaiacol substantially reduces the viscosity of synthesized epoxy monomers due to increased molecular mobility. As shown in Figure 2a, the bio-based epoxy monomer BDEF-EP exhibits a viscosity of 7.36 Pa∙s, which is 42% lower than the conventional E51 epoxy monomer (12.70 Pa∙s). The reduced viscosity endows the synthesized BDEF-EP with superior flow characteristics, facilitating homogeneous mixing and uniform phase distribution during processing. The improved fluidity enables efficient bubble expulsion during both the mixing and curing stages, thereby minimizing void formation and preventing stress concentration defects commonly observed in high-viscosity systems.

The curing kinetics of the BDEF-EP-AA system were studied using DSC at various heating rates. The molar ratio of BDEF-EP to AA was fixed at 1:1, assuming the complete reaction of epoxy and carboxylic acid groups. Components of the BDEF-EP-AA system were blended at room temperature to obtain a homogeneous mixture. Similarly, the E51-AA system was prepared using the same method [22].

The curing exothermic curves obtained at different heating rates of 5, 10, 15, and 20 °C min^−1^ are shown in Figure 2b. A single exothermic peak appeared above 100 °C, indicating that only one ring-opening reaction occurred between the epoxy group and the carboxyl group of the curing agent.

As listed in Table 1, increasing the heating rate enhanced the heat flow rate in the epoxy system and shifted the reaction exothermic peak (*T*_p_) toward higher temperatures. The apparent activation energy (*E*_a_) was determined by fitting the curing kinetics using the Kissinger (Equation (1)) and Ozawa (Equation (2)) equations. The Kissinger method calculates kinetic parameters based on the relationship between the heating rate and the peak temperature of the curing reaction.

(1) $$\ln\left(\frac{\beta }{T_{P}^{2}}\right)=\ln\left(\frac{AR}{E_{a}}\right)-\frac{E_{a}}{RT_{P}}$$

(2) $$\frac{d\ln\beta }{d(\frac{1}{T})}=-1.0518\frac{E_{a}}{R}$$

where *β* is the rate of heating, *T_p_* is the peak temperature, *A* is the predigital factor, and *R* is the molar gas constant 8.314 J/(mol^−1^ K^−1^).

The apparent *E_a_* of the curing process was determined using two methods, with the results shown in Figure 2c and Table 1. The apparent activation energy of bisphenol A-type epoxy resin/anhydride systems is typically in the range of 60–80 kJ mol^−1^. In contrast, the BDEF-EP-AA system exhibited lower activation energies: 56.61 kJ mol^−1^ (Kissinger equation) and 58.94 kJ mol^−1^ (Ozawa equation). The low *E*_a_ is attributed to weaker interchain interactions induced by the incorporation of flexible propyl side chains, which lower the energy barrier for chain segment mobility at elevated temperatures.

Based on the fitting temperatures, the optimal curing procedure for BDEF-EP-AA was established as 120 °C for 4 h followed by 160 °C for 4 h, consistent with the curing conditions for E51-AA. Compared to the FT-IR spectrum of BDEF-EP, the disappearance of the characteristic absorption peak for the epoxy group at 910 cm^−1^ and the emergence of the C=O characteristic absorption peak at 1730 cm^−1^ indicate the formation of a fully crosslinked network in the cured epoxy systems (Figure 2d).

### 3.2. Thermal Stability and Thermomechanical Properties Analysis

**Figure 3:**
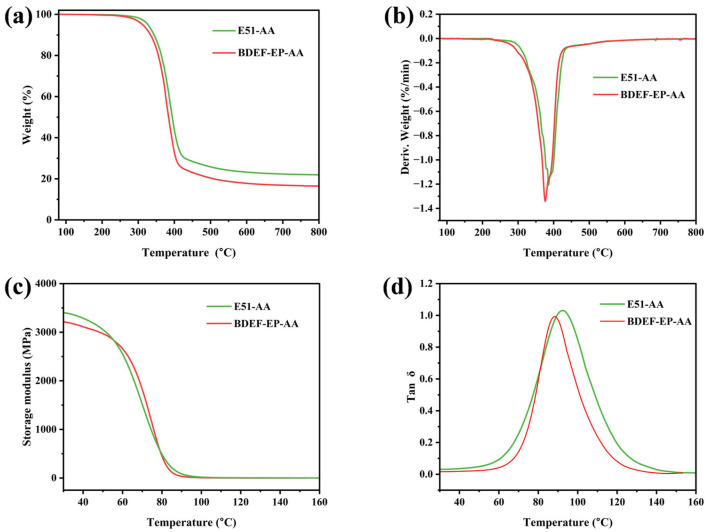
(**a**) Thermogravimetric (TGA), (**b**) derivative thermogravimetric (DTG), (**c**) storage modulus (*E′*), and (**d**) tan delta curves from DMA of BDEF-EP-AA and the reference epoxy resin (E51-AA).

Figure 3 and Table S2 present the thermal stability results of the cured epoxy vitrimers. The TGA curves (Figure 3a) show initial decomposition temperatures of 312 °C for BDEF-EP-AA and 327 °C for E51-AA. The difference in initial decomposition temperature between them is minimal (<15 °C), with both exceeding 300 °C. The DTG curves (Figure 3b) reveal maximum weight loss temperatures of 376 °C for BDEF-EP-AA and 381 °C for E51-AA, indicating comparable thermal decomposition behavior. At 800 °C, BDEF-EP-AA exhibits a char yield of 16.4%, which is lower than that for E51-AA (21.9%), indicating reduced carbon retention capability. Figure 3c,d shows the storage modulus and tan δ of cured epoxy systems as a function of temperature. BDEF-EP-AA exhibits a glass transition temperature (*T*_g_) of 88 °C, which is 4 °C lower than that for E51-AA (92 °C). Moreover, BDEF-EP-AA demonstrates a high storage modulus at 30 °C of 3211 MPa, which is comparable to that of E51-AA (3396 MPa). The lower *T*_g_ and mechanical strength of BDEF-EP-AA, compared to those of E51-AA, are attributed to two main factors. Firstly, the chemical structure of BDEF-EP incorporates flexible propyl side chains, which are absent in E51. While this flexibility reduces prepolymer viscosity, it also increases the molecular volume and chain spacing, consequently diminishing the mechanical strength. Secondly, BDEF-EP has a lower epoxy value than E51. This results in fewer crosslinking points available to form the three-dimensional network during curing, leading to a lower overall crosslink density. This reduced crosslink density is the primary reason for the lower tensile strength of BDEF-EP-AA relative to E51-AA. Nevertheless, BDEF-EP-AA exhibits a comparable glass transition temperature and storage modulus, demonstrating that this bio-based epoxy resin still possesses robust mechanical properties suitable for a wide range of applications. Overall, the synthesized bio-based epoxy resin demonstrates good thermal stability and thermomechanical properties comparable to commercial petroleum-based epoxy resins.

### 3.3. Mechanical Performance Analysis

**Figure 4:**
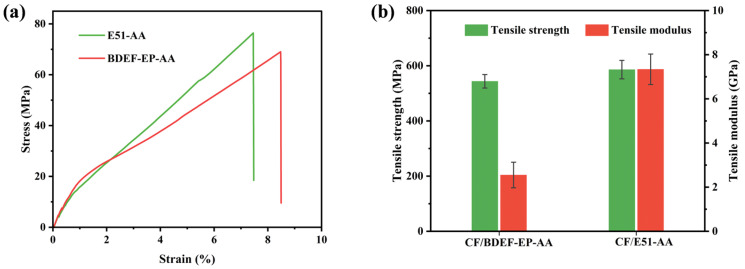
(**a**) Stress–strain curves; (**b**) three-point bending strength and modulus of BDEF-EP-AA and E51-AA.

The tensile properties of BDEF-EP-AA and E51-AA were measured using an electronic universal testing machine, with the results presented in Table 2 and Figure 4. As shown in Figure 4a, BDEF-EP-AA exhibits a slightly lower tensile strength (69.4 MPa) than that of E51-AA (75.0 MPa), while the elongation at break of BDEF-EP-AA is 8.5%, higher than that of E51-AA (7.4%). As quantified in Table S3, BDEF-EP-AA exhibited a superior tensile strength and storage modulus compared to those of other reported bio-based epoxy vitrimers. Notably, BDEF-EP-AA achieved a high storage modulus while maintaining a balance between strength and toughness. This performance is attributed to its dynamic ester exchange crosslinking networks and the migration of flexible propyl side chains, which promote molecular chain entanglement. These features collectively enhance the thermomechanical properties, positioning BDEF-EP-AA as a high-performance bio-based vitrimer.

This difference is attributed to the flexible propyl side chains in BDEF-EP, which can absorb energy during stretching and delay fracture. However, these side chains also reduce intermolecular forces within the crosslinked network under stress, ultimately decreasing mechanical strength due to extension. The flexural strength was evaluated using a three-point bending test. BDEF-EP-AA exhibits a lower flexural strength (105 MPa) than that of E51-AA (128 MPa), indicating reduced rigidity despite improved ductility. However, the flexural modulus shows no significant change. In contrast, the mechanical properties of BDEF-EP-AA and E51-AA show little difference, indicating that bio-based epoxy resins also possess high-strength potential and demonstrating that bio-based materials do not necessitate compromises in performance. Through molecular design and optimized curing processes, a three-dimensional network structure exhibiting a similar crosslink density, chain segment rigidity, and *T*_g_ was achieved. This structural equivalence forms the basis for performance equivalence. Overall, BDEF-EP-AA demonstrates comparable mechanical properties and shows potential as a partial substitute for petroleum-based epoxy resins.

### 3.4. Dynamic Performance

**Figure 5:**
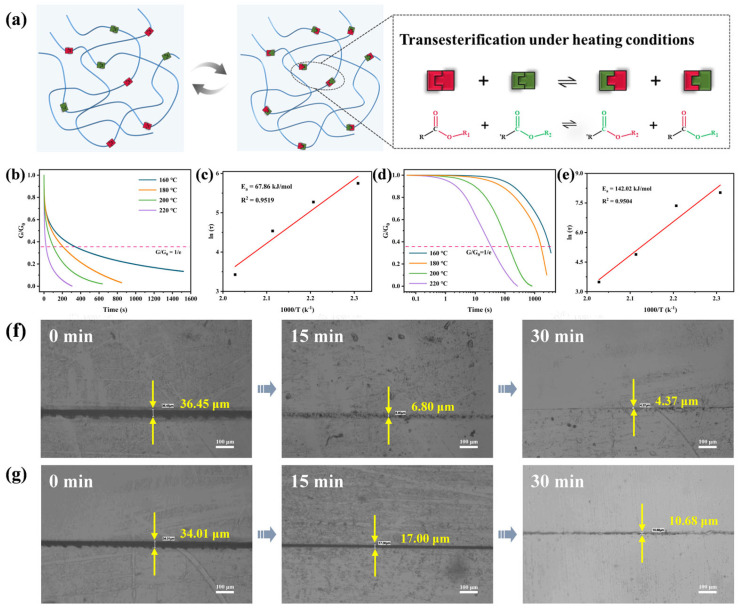
(**a**) Schematic of mechanism for transesterification reactions; normalized stress relaxation curves at different temperatures (160, 180, 200 and 220 °C) for (**b**) BDEF-EP-AA and (**d**) E51-AA; the fitting of relaxation times to the Arrhenius equation for (**c**) BDEF-EP-AA and (**e**) E51-AA; thermal repairing of (**f**) BDEF-EP-AA and (**g**) E51-AA at 200 °C.

Figure 5a schematically illustrates the reversible carboxylic acid ester exchange reaction mechanism. This mechanism indicates that increased chain segment mobility within the crosslinked network at high temperatures facilitates transesterification, leading to polymer topology rearrangement. This process enhances intermolecular interactions and increases network densification [23,24]. Stress relaxation originates from dynamic covalent bond exchange. Specifically, the dynamic behavior of BDEF-EP-AA arises from the synergistic effects of dynamic ester bonds and flexible propyl side chains within its crosslinked network. Unlike conventional polymers, the transesterification enables internal network reorganization and rapid stress relaxation. Thus, for dynamic covalent crosslinked polymers, the stress relaxation time (τ*) is a key parameter for evaluating bond dynamics. According to the Maxwell model for viscoelastic fluids, τ* represents the time required for stress to decay to 1/e (≈37%) of its initial value. Figure 5b displays normalized stress relaxation curves for the bio-based epoxy vitrimer within 160–220 °C. BDEF-EP-AA exhibits rapid relaxation above its *T*_g_, demonstrating accelerated stress relaxation at elevated temperatures. The relaxation time of BDEF-EP-AA decreases from 313 s (160 °C) to less than 30 s (220 °C), whereas that of E51-AA decreases from 3058 s to 50 s under identical conditions (Figure 5d). It is worthy to note that the superior dynamic performance of BDEF-EP-AA exceeds that reported in most of the literature [17,18,25,26,27,28]. Additionally, the stress relaxation time follows Arrhenius temperature dependence. Fitting the logarithmic relaxation time versus temperature (Figure 5c,e) yielded activation energies (*E*_a_) of 67.86 kJ mol^−1^ for BDEF-EP-AA and 142.02 kJ mol^−1^ for E51-AA. A lower *E*_a_ indicated more facile bond exchange reactions requiring less thermal energy, thereby enhancing the reshaping and reprocessing efficiency.

Furthermore, BDEF-EP-AA exhibits a Tv of 34 °C, compared to 118 °C for E51-AA. The significantly lower theoretical Tv of BDEF-EP-AA than that of E51-AA indicates that the flexible propyl groups of BDEF-EP-AA endow the resulting epoxy vitrimer with excellent dynamic properties, such as rapid stress relaxation and a low Ea. Standard scratches (≥30 μm wide) were created on the bio-based glass polymer surfaces using a scalpel blade, followed by thermal activation at 200 °C to trigger dynamic ester bond exchange [17,29]. Scratch width evolution was quantitatively monitored at 15-min intervals using polarized light microscopy. Figure 5f,g depict the scratch width evolution for BDEF-EP-AA and E51-AA, respectively. BDEF-EP-AA achieved a repair efficiency over 80% at 15 min and 90% at 30 min. In contrast, the repair efficiency of E51-AA reached only 50% at 15 min and less than 70% at 30 min. This enhanced self-healing capability is attributed to the flexible propyl groups in BDEF-EP, which facilitate chain segment migration within the crosslinked network and lower the *E*_a_ for dynamic bond exchange, thereby improving the vitrimer’s scratch-repair efficiency.

### 3.5. Chemical Degradation

**Figure 6:**
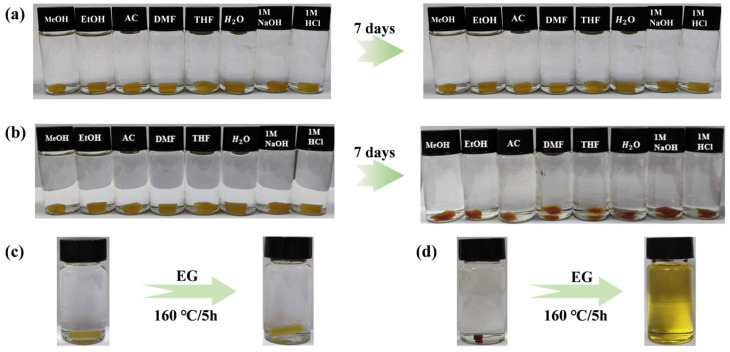
Solvent resistance test for (**a**) BDEF-EP-AA and (**b**) E51-AA at room temperature; chemical degradation in ethylene glycol solvents for (**c**) E51-AA and (**d**) BDEF-EP-AA.

As shown in Figure 6a,b, both the BDEF-EP-AA and E51-AA systems are insoluble in most solvents, demonstrating resistance to organic solvents, acids, and alkalis at ambient temperatures. The chemical stability in solvents is attributed to their densely three-dimensional crosslinked network. However, the controlled cleavage of dynamic bonds allows the epoxy vitrimers to achieve complete degradation in specified conditions. As illustrated in Figure 6c,d, ethylene glycol catalyzes ester bond transesterification through hydroxyl group donation, enabling the complete dissolution of BDEF-EP-AA at 160 °C for 5 h [30,31]. However, E51-AA resists complete degradation under identical conditions. The results indicate that the flexible propyl side chains in BDEF-EP weaken intermolecular forces within the crosslinked network, thereby facilitating the penetration of ethylene glycol solvent and promoting the cleavage of ester bonds. In contrast, the dense structure of E51-AA impedes glycol solvent penetration into its crosslinked network, even under prolonged treatment.

### 3.6. Application in CFRP Composites

**Figure 7:**
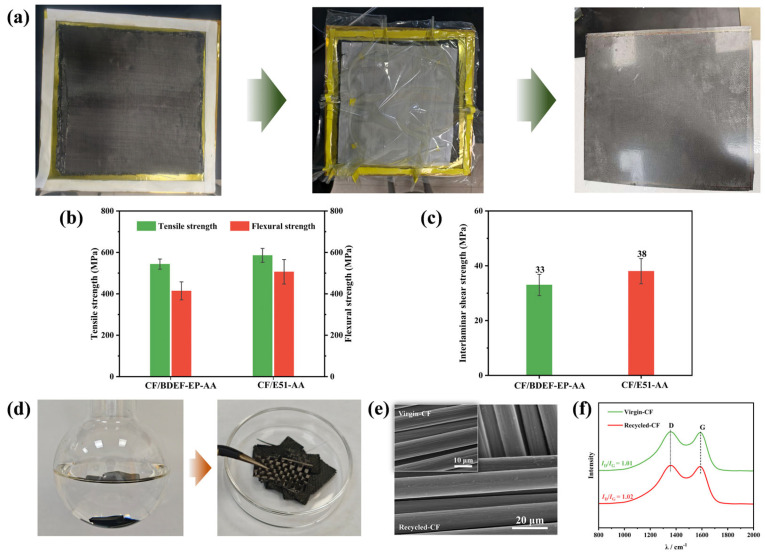
(**a**) CFRP fabrication through hot pressing of sheets prepared by the vacuum introduction molding method; (**b**) tensile strength and flexural strength; (**c**) ILSS of CF/BDEF-EP-AA and CF/E51-AA; (**d**) recycling CF of CF/BDEF-EP-AA in EG at 160 °C/5 h; (**e**) surface morphology of the virgin-CF and recycled-CF tested by SEM; (**f**) Raman spectra of virgin-CF and recycled-CF.

Figure 7a illustrates the CFRP fabrication process via VIM. The low viscosity of BDEF-EP eliminates the need for diluents during CFRP manufacturing, avoiding issues of inhomogeneous resin mixing caused by diluent–epoxy incompatibility. Moreover, the omission of diluents reduces costs and prevents heat-induced surface defects resulting from exothermic reactions during curing. This leads to a significant reduction in bubble and porosity formation, making BDEF-EP a promising candidate for epoxy prepreg production.

The mechanical properties of the CF/BDEF-EP-AA composite were compared with those of the CF/E51-AA composite, with detailed data presented in Table 3. The tensile strength (543.7 MPa), flexural strength (414.2 MPa in Figure 7b), and interlaminar shear strength (ILSS) (33.0 MPa in Figure 7d) of the BDEF-EP-AA/CF composite reached 93%, 82%, and 87% of the values observed for the CF/E51-AA composite, respectively, indicating that the bio-based vitrimer composite possesses favorable mechanical strength.

Based on the degradation study of the bio-based vitrimer, the CFRP samples were cut into small pieces and immersed in a degradation solution, as illustrated in Figure 7e. After treatment at 160 °C for 5 h, the resin matrix was fully dissolved in the glycol-based solvent, allowing for the complete separation of the CF cloth from the composite. The recovered CF fabrics were washed repeatedly with ethanol and dried to constant weight. The SEM analysis (Figure 7f) revealed that the surface of the CF fabrics remained smooth and flat without any damage after degradation. The Raman spectrum (Figure 7f) displayed the characteristic D (~1350 cm^−1^) and G (~1580 cm^−1^) bands. The D band corresponds to sp^3^-hybridized carbon atoms in disordered/defective structures, whereas the G band represents the ideal graphite lattice. The *I*_D_/*I*_G_ intensity ratio indicates the degree of graphitization, with higher values signifying greater structural disorder. The *I*_D_/*I*_G_ ratio of the recovered carbon fibers (1.02) is comparable to that of the virgin fibers, confirming that the degradation preserved both the structural integrity and microstructure of the carbon fibers [32,33].

## 4. Conclusions

In this work, a novel bio-based epoxy vitrimer, BDEF-EP-AA, containing exchangeable ester bonds was synthesized by curing the epoxy monomer BDEF-EP with adipic acid. Owing to the enhanced polymer chain mobility induced by the flexible propyl side chains in BDEF-EP, the dynamic ester exchange reactions within the crosslinking networks were significantly improved, thereby synergistically accelerating the stress relaxation and dynamic properties of the resulting epoxy vitrimer. Furthermore, the flexible propyl side chains reduced the viscosity of BDEF-EP, minimizing the need for diluents in resin formulations and thereby facilitating CFRP manufacturing process. Consequently, the BDEF-EP-AA system exhibited rapid stress relaxation, efficient scratch healing, and controlled chemical degradability. Moreover, carbon fiber-reinforced BDEF-EP-AA composite was fabricated using a VTM method. The dynamic ester bonds enabled the complete degradation of resin matrix in ethylene glycol and the recovery of high-value CFs with original morphology and structure. Therefore, this study establishes a streamlined strategy for designing bio-based epoxy vitrimers and CFRP composites with enhanced dynamic properties without significantly compromising mechanical properties.

## Figures and Tables

**Table 1:** Peak temperature and activation energy of the curing of BDEF-EP-AA.

Heating Rate (°C min^−1^)	*T_p_* (°C)	*E_a_* Fitted by Kissinger (kJ·mol^−1^)	R^2^	*E_a_* Fitted by Ozawa (kJ·mol^−1^)	R^2^
5	115.3	56.61	0.9944	58.94	0.9939
10	120.5				
15	126.1				
20	133.8				

**Table 2:** Mechanical properties of BDEF-EP-AA and E51-AA.

Sample	Tensile Strength (MPa)	Young’s Modulus (MPa)	Elongation (%)	Flexural Strength (MPa)	Flexural Modulus (GPa)
BDEF-EP-AA	69.4 ± 1.3	1290.2 ± 39.1	8.5 ± 0.6	105 ± 7.9	2.2 ± 0.6
E51-AA	75.0 ± 1.8	1350.1 ± 45.7	7.4 ± 0.8	128 ± 9.4	2.6 ± 0.8

**Table 3:** Mechanical properties of CF/BDEF-EP-AA and CF/E51-AA.

Sample	Tensile Strength (MPa)	Flexural Strength (MPa)	ILSS (MPa)
CF/BDEF-EP-AFD	543.7 ± 28.4	414.2 ± 43.4	33.0 ± 3.8
CF/E51-AA	585.9 ± 34.9	506.6 ± 59.0	38.0 ± 4.5

## Data Availability

Data is contained within the article or Supplementary Material. The original contributions presented in this study are included in the article/Supplementary Material. Further inquiries can be directed to the corresponding author.

## Supplementary Materials

The following supporting information can be downloaded at https://www.mdpi.com/article/10.3390/polym17172387/s1: Table S1: Epoxy vitrimer formulations of BDEF-EP-AA and E51-AA. Table S2: Thermal stability and thermomechanical properties of BDEF-EP-AA and E51-AA. Table S3: Comparative mechanical strength and modulus for bio-based epoxy resins. Refs. [17,21,34,35,36,37,38,39] are cited in the Supplementary Materials.

## References

[1] Jin F.-L., Li X., Park S.-J. (2015). Synthesis and application of epoxy resins: A review. J. Ind. Eng. Chem..

[2] Meng J., Chen P., Yang R., Dai L., Yao C., Fang Z., Guo K. (2021). Thermal stable honokiol-derived epoxy resin with reinforced thermal conductivity, dielectric properties and flame resistance. Chem. Eng. J..

[3] Post W., Susa A., Blaauw R., Molenveld K., Knoop R.J.I. (2020). A Review on the Potential and Limitations of Recyclable Thermosets for Structural Applications. Polym. Rev..

[4] Jensen J.P., Skelton K. (2018). Wind turbine blade recycling: Experiences, challenges and possibilities in a circular economy. Renew. Sustain. Energy Rev..

[5] Shundo A., Yamamoto S., Tanaka K. (2022). Network Formation and Physical Properties of Epoxy Resins for Future Practical Applications. JACS Au.

[6] Zhao X., Long Y., Xu S., Liu X., Chen L., Wang Y.-Z. (2023). Recovery of epoxy thermosets and their composites. Mater. Today.

[7] Czarny-Krzymińska K., Krawczyk B., Szczukocki D. (2023). Bisphenol A and its substitutes in the aquatic environment: Occurrence and toxicity assessment. Chemosphere.

[8] Andújar N., Gálvez-Ontiveros Y., Zafra-Gómez A., Rodrigo L., Álvarez-Cubero M.J., Aguilera M., Monteagudo C., Rivas A. (2019). Bisphenol A Analogues in Food and Their Hormonal and Obesogenic Effects: A Review. Nutrients.

[9] Moon M.K., Jeong I.-K., Jung Oh T., Ahn H.Y., Kim H.H., Park Y.J., Jang H.C., Park K.S. (2015). Long-term oral exposure to bisphenol A induces glucose intolerance and insulin resistance. J. Endocrinol..

[10] Lucherelli M.A., Duval A., Avérous L. (2022). Biobased vitrimers: Towards sustainable and adaptable performing polymer materials. Prog. Polym. Sci..

[11] Zhang C., Xue J., Yang X., Ke Y., Ou R., Wang Y., Madbouly S.A., Wang Q. (2022). From plant phenols to novel bio-based polymers. Prog. Polym. Sci..

[12] Mashouf Roudsari G., Mohanty A.K., Misra M. (2017). Green Approaches To Engineer Tough Biobased Epoxies: A Review. ACS Sustain. Chem. Eng..

[13] Xin J., Li M., Li R., Wolcott M.P., Zhang J. (2016). Green Epoxy Resin System Based on Lignin and Tung Oil and Its Application in Epoxy Asphalt. ACS Sustain. Chem. Eng..

[14] Kamarulzaman S., Png Z.M., Lim E.Q., Lim I.Z.S., Li Z., Goh S.S. (2023). Covalent adaptable networks from renewable resources: Crosslinked polymers for a sustainable future. Chem.

[15] Montarnal D., Capelot M., Tournilhac F., Leibler L. (2011). Silica-Like Malleable Materials from Permanent Organic Networks. Science.

[16] Ruiz de Luzuriaga A., Martin R., Markaide N., Rekondo A., Cabañero G., Rodríguez J., Odriozola I. (2016). Epoxy resin with exchangeable disulfide crosslinks to obtain reprocessable, repairable and recyclable fiber-reinforced thermoset composites. Mater. Horiz..

[17] Liu T., Hao C., Wang L., Li Y., Liu W., Xin J., Zhang J. (2017). Eugenol-Derived Biobased Epoxy: Shape Memory, Repairing, and Recyclability. Macromolecules.

[18] Chen M., Zhou L., Wu Y., Zhao X., Zhang Y. (2019). Rapid Stress Relaxation and Moderate Temperature of Malleability Enabled by the Synergy of Disulfide Metathesis and Carboxylate Transesterification in Epoxy Vitrimers. ACS Macro Lett..

[19] Chen J.-H., Lu J.-H., Pu X.-L., Chen L., Wang Y.-Z. (2022). Recyclable, malleable and intrinsically flame-retardant epoxy resin with catalytic transesterification. Chemosphere.

[20] Liu H., Sun Z., Wei L., Liu Y., Zhou S., Ge Q., Liu C., Li X. (2023). Double-dynamic crosslinked epoxy vitrimer resin prepared using transesterification and dynamic disulfide bonds: High-performance, degradable, self-healing, environment-friendly. Polym. Test..

[21] Dong K., Zhao D., Pang Y., Liu B., Liu Q., Mu T., Zhao C. (2025). Multiple-reprocessable guaiacol-derived epoxy vitrimer with disulfide crosslinks and closed-loop recycling of carbon fiber-reinforced composites. Chem. Eng. J..

[22] Zeng R.-T., Wu Y., Li Y.-D., Wang M., Zeng J.-B. (2017). Curing behavior of epoxidized soybean oil with biobased dicarboxylic acids. Polym. Test..

[23] Kumar A., Connal L.A. (2023). Biobased Transesterification Vitrimers. Macromol. Rapid Commun..

[24] Ye G., Huo S., Wang C., Zhang Q., Wang H., Song P., Liu Z. (2024). Strong yet Tough Catalyst-Free Transesterification Vitrimer with Excellent Fire-Retardancy, Durability, and Closed-Loop Recyclability. Small.

[25] Zhao X.-L., Liu Y.-Y., Weng Y., Li Y.-D., Zeng J.-B. (2020). Sustainable Epoxy Vitrimers from Epoxidized Soybean Oil and Vanillin. ACS Sustain. Chem. Eng..

[26] Schenk V., De Calbiac J., D’Elia R., Olivier P., Labastie K., Destarac M., Guerre M. (2024). Epoxy Vitrimer Formulation for Resin Transfer Molding: Reactivity, Process, and Material Characterization. ACS Appl. Polym. Mater..

[27] Liu Y.-Y., He J., Li Y.-D., Zhao X.-L., Zeng J.-B. (2020). Biobased epoxy vitrimer from epoxidized soybean oil for reprocessable and recyclable carbon fiber reinforced composite. Compos. Commun..

[28] Hu Y., Tong S., Hu L., Zhang M., Huang Q., Sha Y., Jia P., Zhou Y. (2023). Molecularly engineered cardanol derived epoxy vitrimers based on dynamic disulfide and dynamic ester exchanges with desirable dynamic response, degradability, and recyclability. Chem. Eng. J..

[29] Wang M., Gao H., Wang Z., Mao Y., Yang J., Wu B., Jin L., Zhang C., Xia Y., Zhang K. (2022). Rapid self-healed vitrimers via tailored hydroxyl esters and disulfide bonds. Polymer.

[30] Leung W.H., Leitao E.M., Verbeek C.J.R. (2025). Polyester transesterification through reactive blending and its applications: A comprehensive review. Polymer.

[31] Xia J., Li S., Gao R., Zhang Y., Wang L., Ye Y., Cao C., Xue H. (2024). Bio-Based Epoxy Vitrimers with Excellent Properties of Self-Healing, Recyclability, and Welding. Polymers.

[32] Xu P., Li J., Ding J. (2013). Chemical recycling of carbon fibre/epoxy composites in a mixed solution of peroxide hydrogen and N,N-dimethylformamide. Compos. Sci. Technol..

[33] Li W., Xiao L., Huang J., Wang Y., Nie X., Chen J. (2022). Bio-based epoxy vitrimer for recyclable and carbon fiber reinforced materials: Synthesis and structure-property relationship. Compos. Sci. Technol..

[34] Dong K., Tang S., Zhao D., Pang Y., Zhao C. (2024). Vanillin-derived bio-based epoxy resins containing dual dynamic Schiff base and disulfide bonds with reprocessability and degradability. Polym. Degrad. Stab..

[35] Tang S., Lin H., Dong K., Zhang J., Zhao C. (2023). Closed-loop recycling and degradation of guaiacol-based epoxy resin and its carbon fiber reinforced composites with S-S exchangeable bonds. Polym. Degrad. Stab..

[36] Zhao S., Abu-Omar M.M. (2018). Recyclable and Malleable Epoxy Thermoset Bearing Aromatic Imine Bonds. Macromolecules.

[37] Hu Y., Tong S., Sha Y., Yu J., Hu L., Huang Q., Jia P., Zhou Y. (2023). Cardanol-based epoxy vitrimer/carbon fiber composites with integrated mechanical, self-healing, reprocessable, and welding properties and degradability. Chem. Eng. J..

[38] Zhao C., Huang G., Zhang H., Xie H., Sha F., Feng L., Cui J., Li X., Wang M., Bao F. (2024). High-homogeneous recyclable self-cured epoxy resins based on imine. Chem. Eng. J..

[39] Zhao S., Abu-Omar M.M. (2019). Catechol-Mediated Glycidylation toward Epoxy Vitrimers/Polymers with Tunable Properties. Macromolecules.

