# Supplementary material for: Repairable, Degradable and Recyclable Carbon Fiber-Reinforced Bio-Based Epoxy Vitrimer Composites Enabled by Facile Transesterification
Source: Polymers (Basel). 2025 Aug 31;17(17):2387. doi: 10.3390/polym17172387 (PMC12431544; doi:10.3390/polym17172387)
Supplement: Supplementary file 1 [file polymers-17-02387-s001.zip › polymers-3832378-supplementary.pdf]

Supporting Information

Repairable, Degradable, and Recyclable Carbon Fiber-Reinforced Bio-Based Epoxy Vitrimer Composites Enabled by Facile Transesterification

Haidan Lin [1, 2], Kai Dong [1], Jingyao Luan [2], Chenggang Li [2], Di Zhao [1], Chengji Zhao [1], Xuefeng Li [1*]

[1]Key Laboratory of High Performance Plastics, Ministry of Education, College of Chemistry, Jilin University, Changchun 130012, P R China;

[2]Electric Power Research Institute, State Grid Jilin Electric Power Co., Ltd., Changchun 130012, PR China;

*Correspondence: xuefengli@jlu.edu.cn; Tel.: +86 431 85168870

Table S1. Epoxy vitrimer formulations of BDEF-EP-AA and E51-AA.

	BDEF-EP (g)	E51 (g)	AA (g)	$Zn(acac)_2$ (g)
BDEF-EP-AA	5.0	0	1.6	0.28
E51-AA	0	5.0	1.8	0.33

Table S2. Thermal stability and thermomechanical properties of BDEF-EP-AA and E51-AA.

	$T_{d5\%}$ (°C)	$T_{d10\%}$ (°C)	Residue at 800 °C (%)	T_g (°C)	Storage modulus (MPa)
BDEF-EP-AA	312	334	16.4	88 ± 2.6	3211 ± 78
E51-AA	327	344	21.9	92 ± 3.1	3396 ± 86

Table S3. Comparative mechanical strength and modulus for bio-based epoxy resins.

Sample	Storage modulus (MPa)	Tensile strength (MPa)	***Ref.***
BDEF-EP-AA	3211	69.4	**This work**
BDEF-EP-AFD	2400	42	**[1]**
BVF-EP/AFD(R=0.5)	2528	28	**[2]**
BGF-EP-AFD	1414	54	**[3]**
EN-VAN-AP	2004	46	**[4]**
EN-BPA	1925	45	
EDCS-HP3	1351.2	28.7	**[5]**
EDCS-HP5	1674.4	20.5	
EuEP/SA with R= 1:0.5	2250	25	**[6]**
VEP-MeHHPA	2205	40.1	**[7]**
$E1_{36}E3_{64}$	2600	25.1	**[8]**

[1] K. Dong, D. Zhao, Y. Pang, B. Liu, Q. Liu, T. Mu, C. Zhao, Multiple-reprocessable guaiacol-derived epoxy vitrimer with disulfide crosslinks and closed-loop recycling of carbon fiber-reinforced composites, Chem. Eng. J. 2025, 508, 160754.

[2] K. Dong, S. Tang, D. Zhao, Y. Pang, C. Zhao, Vanillin-derived bio-based epoxy resins containing dual dynamic Schiff base and disulfide bonds with reprocessability and degradability, Polym. Degrad. Stab. 2024, 230, 111077.

[3] S. Tang, H. Lin, K. Dong, J. Zhang, C. Zhao, Closed-loop recycling and degradation of guaiacol-based epoxy resin and its carbon fiber reinforced composites with S-S exchangeable bonds, Polym. Degrad. Stab. 2023, 210, 110298.

[4] Zhao, S.; Abu-Omar, M.M. Recyclable and Malleable Epoxy Thermoset Bearing Aromatic Imine Bonds. Macromolecules 2018, 51, 9816-9824.

[5] Hu, Y.; Tong, S.; Sha, Y.; Yu, J.; Hu, L.; Huang, Q.; Jia, P.; Zhou, Y. Cardanol-based epoxy vitrimer/carbon fiber composites with integrated mechanical, self-healing, reprocessable, and welding properties and degradability. Chem. Eng. J. 2023, 471, 144633.

[6] Liu, T.; Hao, C.; Wang, L.; Li, Y.; Liu, W.; Xin, J.; Zhang, J. Eugenol-Derived Biobased Epoxy: Shape Memory, Repairing, and Recyclability. Macromolecules 2017, 50, 8588-8597.

[7] Zhao, C.; Huang, G.; Zhang, H.; Xie, H.; Sha, F.; Feng, L.; Cui, J.; Li, X.; Wang, M.; Bao, F.; et al. High-homogeneous recyclable self-cured epoxy resins based on imine. Chem. Eng. J. 2024, 501, 157047.

[8] Zhao, S.; Abu-Omar, M.M. Catechol-Mediated Glycidylation toward Epoxy Vitrimers/Polymers with Tunable Properties. Macromolecules 2019, 52, 3646-3654.
